# Oscillatory Pattern of Sympathetic Nerve Bursts Is Associated With Baroreflex Function in Heart Failure Patients With Reduced Ejection Fraction

**DOI:** 10.3389/fnins.2021.669535

**Published:** 2021-08-31

**Authors:** Edgar Toschi-Dias, Nicola Montano, Eleonora Tobaldini, Patrícia F. Trevizan, Raphaela V. Groehs, Ligia M. Antunes-Correa, Thais S. Nobre, Denise M. Lobo, Allan R. K. Sales, Linda M. Ueno-Pardi, Luciana D. N. J. de Matos, Patrícia A. Oliveira, Ana Maria F. W. Braga, Maria Janieire N. N. Alves, Carlos E. Negrão, Maria Urbana P. B. Rondon

**Affiliations:** ^1^Instituto do Coração (InCor), Hospital das Clinicas HCFMUSP, Faculdade de Medicina, Universidade de São Paulo, São Paulo, Brazil; ^2^Department of Internal Medicine, Fondazione IRCSS Ca’ Granda, Ospedale Maggiore Policlínico, Milan, Italy; ^3^Department of Clinical Sciences and Community Health, University of Milan, Milan, Italy; ^4^School of Physical Education and Sport, University of São Paulo, São Paulo, Brazil

**Keywords:** heart failure, oscillatory pattern, sympathetic nervous system, baroreflex control, cardiovascular variabilities

## Abstract

Sympathetic hyperactivation and baroreflex dysfunction are hallmarks of heart failure with reduced ejection fraction (HFrEF). However, it is unknown whether the progressive loss of phasic activity of sympathetic nerve bursts is associated with baroreflex dysfunction in HFrEF patients. Therefore, we investigated the association between the oscillatory pattern of muscle sympathetic nerve activity (LF_MSNA_/HF_MSNA_) and the gain and coupling of the sympathetic baroreflex function in HFrEF patients. In a sample of 139 HFrEF patients, two groups were selected according to the level of LF_MSNA_/HF_MSNA_ index: (1) Lower LF_MSNA_/HF_MSNA_ (lower terciles, *n* = 46, aged 53 ± 1 y) and (2) Higher LF_MSNA_/HF_MSNA_ (upper terciles, *n* = 47, aged 52 ± 2 y). Heart rate (ECG), arterial pressure (oscillometric method), and muscle sympathetic nerve activity (microneurography) were recorded for 10 min in patients while resting. Spectral analysis of muscle sympathetic nerve activity was conducted to assess the LF_MSNA_/HF_MSNA_, and cross-spectral analysis between diastolic arterial pressure, and muscle sympathetic nerve activity was conducted to assess the sympathetic baroreflex function. HFrEF patients with lower LF_MSNA_/HF_MSNA_ had reduced left ventricular ejection fraction (26 ± 1 vs. 29 ± 1%, *P* = 0.03), gain (0.15 ± 0.03 vs. 0.30 ± 0.04 a.u./mmHg, *P* < 0.001) and coupling of sympathetic baroreflex function (0.26 ± 0.03 vs. 0.56 ± 0.04%, *P* < 0.001) and increased muscle sympathetic nerve activity (48 ± 2 vs. 41 ± 2 bursts/min, *P* < 0.01) and heart rate (71 ± 2 vs. 61 ± 2 bpm, *P* < 0.001) compared with HFrEF patients with higher LF_MSNA_/HF_MSNA_. Further analysis showed an association between the LF_MSNA_/HF_MSNA_ with coupling of sympathetic baroreflex function (*R* = 0.56, *P* < 0.001) and left ventricular ejection fraction (*R* = 0.23, *P* = 0.02). In conclusion, there is a direct association between LF_MSNA_/HF_MSNA_ and sympathetic baroreflex function and muscle sympathetic nerve activity in HFrEF patients. This finding has clinical implications, because left ventricular ejection fraction is less in the HFrEF patients with lower LF_MSNA_/HF_MSNA_.

## Introduction

Heart failure is a complex syndrome and considered the leading cause of hospitalization in patients over the age of 60 years, which accounts for about 30–40% of the mortality of these patients (Ponikowski et al., 2016). According to cardiac function, heart failure (HF) patients are currently classified as follows: (i) with preserved ejection fraction, (ii) with mid-range ejection fraction; and (iii) with reduced ejection fraction (HFrEF). It is also known that neurohumoral excitation is a hallmark of HFrEF and an independent predictor of mortality in patients suffering with this syndrome (Barretto et al., 2009).

HFrEF patients have increased sympathetic activity as shown by the enhanced sympathetic nerve discharges (Barretto et al., 2009; Triposkiadis et al., 2009), and a progressive loss of phasic activity of sympathetic nerve bursts (van de Borne et al., 1997). It is known that the sympathetic nervous system influence on cardiovascular control depends not only on its tonic but also its phasic activity (i.e., modulation). Thus, both the frequency and the intensity of the sympathetic nerve discharge determine a pattern of oscillation that influences the efficiency of sympathetic effector response (Toschi-Dias et al., 2013). This modulation of sympathetic nerve bursts may be evaluated by the balance between the spectral components of low (LF) and high frequency (HF) sympathetic nerve activity and represents the intrinsic behavior of sympathetic nervous system functioning (van de Borne et al., 1997; Toschi-Dias et al., 2013).

In healthy individuals, spontaneous fluctuations in heart rate (HR), blood pressure (BP), and muscle sympathetic nerve activity (MSNA) are strongly coupling in the LF band both at rest and during physiological maneuvers (e.g., orthostatic stress) (Malliani et al., 1991; Stauss et al., 1998; Furlan et al., 2000). Based on neuromodulation approaches, Stauss et al. (1998) demonstrated that vascular tonus is modulated by sympathetic nerve stimulations in the frequency range between 0.07 and 0.10 Hz in healthy subjects. However, sympathetic nerve stimulation reduced blood flow when the stimulation frequency was in the HF range (i.e., > 0.20 Hz). These findings indicate that peripheral sympathetic transmission to the vascular bed of humans acts as a low-pass filter with a cut-off frequency above 0.10 Hz (Stauss et al., 1998).

On the other hand, the increase of burst frequency with a shift from LF fluctuations toward HF bands (∼0.20 Hz) are linked with a marked reduction in the linear relationship between systolic arterial pressure (SAP) and MSNA oscillation in the presyncope phase during a tilt test maneuver (Kamiya et al., 2005; Barbic et al., 2015). These data demonstrate the physiological relevance of spectral density and coupling between cardiovascular variability parameters, because to induce an optimal vasomotor response, the tonic and phasic activities of the sympathetic firings must occur at ∼0.10 Hz for appropriate vasoconstriction (Pagani et al., 1997; Stauss et al., 1998; Furlan et al., 2000; Kamiya et al., 2005; Barbic et al., 2015).

A neural mechanism of interest is the baroreflex control, which exerts a major inhibitory influence on sympathetic outflow (Joyner et al., 2010). This autonomic reflex control modulates, at least in part, the tonus and the oscillatory pattern of MSNA (LF_MSNA_/HF_MSNA_) (Groehs et al., 2015). The effectiveness of the sympathetic baroreflex depends on its magnitude and coupling responses (Sunagawa et al., 2001). Curiously, baroreflex dysfunction is also a hallmark of HFrEF. The clinical implication of this knowledge is based on the fact that both baroreflex dysfunction and increased MSNA are associated with a poor prognosis in patients with HFrEF sympathetic nerve discharges (Grassi et al., 2004; Barretto et al., 2009), and a progressive loss of phasic activity of sympathetic nerve bursts. However, it is unknown whether a lower LF_MSNA_/HF_MSNA_ can potentiate the sympathetic baroreflex dysfunction in HFrEF patients. In the present study, we tested the hypothesis that HFrEF patients with lower LF_MSNA_/HF_MSNA_ would have an exacerbated hyperadrenergic state compared with HFrEF patients with higher LF_MSNA_/HF_MSNA_. In addition, we sought to determine whether there would be an association between LF_MSNA_/HF_MSNA_ and the gain and coupling of sympathetic baroreflex function in these patients.

## Materials and Methods

This study was approved by the Scientific Commission of the Heart Institute (InCor), University of São Paulo Medical School (#3946/13/071) and Human Subject Protection Committee of the Clinical Hospital, University of São Paulo, Medical School (# 22255213.2.0000.0068). Signed informed consent was obtained from all patients during the screening visit.

### Subjects

Patients were selected from a database of randomized studies performed at the Unit of Cardiovascular Rehabilitation and Exercise Physiology of the Heart Institute (InCor), University of São Paulo Medical School. Initially, 139 HFrEF patients, age ranging from 30 to 65 years, New York Heart Association functional class II to III, left ventricular ejection fraction (LVEF) < 40%, and peak oxygen uptake (VO_2_) < 20 ml.kg^–1^.min^–1^ were included in the study. The exclusion criteria were recent myocardial infarction or unstable angina (<3 months), HFrEF duration (<3 months), and permanent pacemaker dependence. According to the level of the LF_MSNA_/HF_MSNA_ index, patients were placed into two groups: (1) Lower LF_MSNA_/HF_MSNA_ (lower terciles < 0.46, *n* = 46, aged 53 ± 1 y) and (2) Higher LF_MSNA_/HF_MSNA_ (upper terciles > 1.24, *n* = 47, aged 52 ± 2 y).

### Cardiac Function

According to international guidelines, LVEF was evaluated with two-dimensional echocardiography according to the Simpson method (IE33, Philips Medical Systems, Andover, MA) (Ponikowski et al., 2016).

### Functional Capacity

To assess functional capacity, all patients underwent cardiopulmonary exercise testing as previously described (Martinez et al., 2011) on a braked cycle ergometer, using a ramp protocol with work rate increments of 10, 15, or 20 W every minute at 60 rpm up to exhaustion. Peak value of oxygen uptake (VO_2_ peak) was averaged from the last 30 s interval and was considered the maximal exercise capacity (Martinez et al., 2011).

### Muscle Sympathetic Nerve Activity

MSNA was recorded from the peroneal nerve using the microneurography technique (Vallbo et al., 1979; Toschi-Dias et al., 2013). In brief, multiunit postganglionic muscle sympathetic nerve recordings were made using a tungsten microelectrode (tip diameter 5–15 μm). The signals were amplified by a factor of 50–100K and band-pass filtered (0.7–2 KHz). For recordings and analysis, nerve activity was rectified and integrated with time constant at 0.1 s to obtain a mean voltage display of MSNA. In the present study, the tonic activity of the MSNA was evaluated through a time-domain analysis and expressed as burst frequency (i.e., bursts/min) and burst incidence (i.e., burst per 100 heartbeats) (Vallbo et al., 1979; Toschi-Dias et al., 2013). The phasic activity of MSNA was evaluated by a frequency domain analysis (i.e., power spectrum analysis of MSNA variability) and refers solely to the oscillatory pattern of the post-ganglionic sympathetic firing (Toschi-Dias et al., 2013).

### Arterial Pressure, HR, and Respiratory Rate

Arterial pressure was monitored non-invasively by a finger photoplethysmography device (Finapres 2,300, Ohmeda, Englewood, CO) on a beat-to-beat basis. Simultaneously, HR was monitored through lead II of the ECG and respiratory rate was monitored with a piezoelectric thoracic belt (Pneumotrace II, model 1132, UFI, CA) placed around the upper abdomen.

### Experimental Protocol

On the day of the experiment, all patients abstained from caffeine or other types of stimulants for 12 h. The protocol experiment was performed at approximately 8:00 AM, with the patients in a supine position in a quiet air-conditioned room (22–24°C). After obtaining an adequate sympathetic nerve recording site in the leg and after stabilization of the autonomic and cardiovascular variables, baseline recordings of arterial pressure, HR, MSNA, and respiratory rate were taken for 10 min.

### Autonomic Control

After synchronization among signals, specific software (HeartScope II; AMPS-LLC, NY) was used by a trained investigator (ET-D) to process the MSNA, ECG, arterial pressure, and respiratory activity signals and to extract the time series of MSNA, R-R interval (RRi), systolic and diastolic arterial pressure (SAP and DAP, respectively), and respiration on a beat-to-beat basis. The sympathetic bursts were sampled once per cardiac cycle synchronously with the peak of the R-wave of ECG and automatically detected considering an amplitude threshold of 30% of baseline and rejection ratio of 3%. The maximum and minimum values of arterial pressure inside the *i*-th heart period were defined as SAP and DAP values, respectively, where *i* is the cardiac beat counter. Thereafter, the beat-to-beat variability of MSNA, RRi, systolic and diastolic arterial pressure (SAP and DAP, respectively), and respiratory activity were analyzed by an autoregressive frequency domain approach. On stationary segments of 200–300 beats, autoregressive spectral decomposition of times series were calculated based on the Levinson–Durbin recursion with the order of the model chosen according to Akaike’s criterion. This procedure automatically quantifies the center frequency and the power spectral density of oscillatory components in very low (VLF: 0.003–0.04 Hz), low (LF: 0.04–0.15 Hz), and high frequency (HF: 0.15–0.40 Hz) ranges in absolute (abs.) values as well as in normalized units (n.u.). However, only the normalized units were used of the LF and HF components of RRi and MSNA variability due to the variances in data. According to the international guidelines, the RRi spectral component quantified in the LF band representing cardiac sympathetic modulation predominance, and the HF band synchronized with respiration, representing cardiac parasympathetic modulation (Malliani et al., 1991; Montano et al., 1994; Task Force, 1996). Spectral densities of SAP and DAP variability were quantified only in the LF band, indexes of the vasomotor sympathetic modulation (Malliani et al., 1991), because the quantified oscillatory component in the HF band did not represent an autonomic index (Bertram et al., 2000). Finally, due to central medullary sympathetic premotor oscillatory circuits and/or baroreflex resonance, the spectral density in the LF range of MSNA variability reflects the profile of oscillation of sympathetic modulation associated with 0.10-Hz rhythm, whereas spectral density in HF range reflects the marked influence of the central respiratory drive on medullary sympathetic premotor neurons (Malliani et al., 1991; Furlan et al., 2000; Montano et al., 2009). Furthermore, the LF/HF ratio of RRi and MSNA were calculated for estimation of the cardiac sympatho-vagal balance (LF_*RRi*_/HF_*RRi*_) and oscillatory pattern of MSNA (LF_MSNA_/HF_MSNA_), respectively (Martinez et al., 2011; Toschi-Dias et al., 2013; Groehs et al., 2015).

## Sympathetic Baroreflex Control

To evaluate sympathetic baroreflex function, transfer function analysis by means of the bivariate autoregressive model was used with model order fixed to 10. As previously described in detail (Pinna et al., 2002; Bari et al., 2019), this procedure enables quantification of the gain, phase shift, and coherence of transfer function between two signals (e.g., MSNA and DAP) in a frequency range. Briefly, the transfer function was estimated as the ratio of the cross-spectrum computed from input signal (i.e., DAP) to the output signal (i.e., MSNA) to the power spectrum of the input signal. Thus, the gain of sympathetic baroreflex function measures the intensity of the response of the MSNA per unit of spontaneous change of the DAP, being expressed in a.u./mmHg (Pinna et al., 2002; Toschi-Dias et al., 2013; Bari et al., 2019). The phase shift estimated the delay between the oscillations in both variability signals (the SAP changes precede MSNA changes) and was accepted when it was between 0 and -π (radians). The squared coherence function of MSNA and DAP signals was estimated as a ratio of the squared cross-spectrum modulus to the product of the densities spectra of the input and output signals. This function ranged between 0 and 1, with 0 indicating null correlation and 1 maximum correlation. In the present study, the squared coherence index represents the level of neurovascular coupling between MSNA and DAP. The phase shift measures the time lag or lead between the signals. We calculated these indices of the transfer function in the frequency where the coherence peaked at the maximum value within the LF range (Pinna et al., 2002; Toschi-Dias et al., 2013; Bari et al., 2019).

### Statistical Analysis

The data are presented as mean ± standard error. A chi-square (χ^2^) test was used to assess categorical data differences. For each continuous or discrete variable, Lèvene and Kolmogorov–Smirnov tests were used to assess the homogeneity and normality of distribution, respectively. Demographic data and baseline physical characteristics and hemodynamic and autonomic data were compared using Student’s *t*-tests or Mann–Whitney tests when appropriate. Spearman correlation was used to test the association between LF_MSNA_/HF_MSNA_, LVEF, VO_2_, gain and coherence of sympathetic baroreflex function. Probability values of *P* < 0.05 were considered statistically significant.

## Results

Baseline characteristics of patients with HFrEF are shown in Table 1. Age, body mass index (BMI), sex, mean BP, and HFrEF etiology were similar between groups. However, LVEF and VO_2_ were significantly lower and HR and proportion of patients using anticoagulant were significantly higher in the group with lower LF_MSNA_/HF_MSNA_ compared with the group with higher LF_MSNA_/HF_MSNA_.

**TABLE 1:** Baseline characteristics of HfrEF patients with lower and higher oscillatory pattern of MSNA.

	Lower LF_MSNA_/HF_MSNA_	Higher LF_MSNA_/HF_MSNA_	*P*
N	46	47	
Age, y	53 ± 1	52 ± 2	0.91
BMI, kg/m^2^	26 ± 1	27 ± 1	0.32
**Sex**			
Male, n (%)	33 (72)	38 (81)	0.30
Female, n (%)	13 (28)	9 (19)	
LVEF,%	26 ± 1	29 ± 1	0.03
VO_2_ peak, ml.kg^–1^.min^–1^	16 ± 1	18 ± 1	0.03
MAP, mmHg	89 ± 2	86 ± 2	0.89
HR, bpm	72 ± 2	63 ± 2	< 0.001
**HFrEF etiology**			
Idiopathic, n (%)	21 (46)	18 (38)	0.47
Ischaemic, n (%)	14 (30)	15 (32)	0.88
Hypertensive, n (%)	4 (9)	8 (17)	0.23
Chagasic, n (%)	7 (15)	6 (13)	0.73
**Medications**			
β-Blocker, n (%)	44 (96)	45 (96)	0.98
ACEI/ARA, n (%)	44 (96)	44 (94)	0.66
Diuretics, n (%)	42 (91)	46 (98)	0.16
Anticoagulant, n (%)	17 (36)	7 (15)	0.02
Digitalis, n (%)	9 (20)	10 (22)	0.84
Statins, n (%)	19 (41)	22 (47)	0.59

*Values are means ± SE; HFrEF, heart failure with reduced ejection fraction; LF_MSNA_/HF_MSNA_, oscillatory pattern of MSNA; BMI, body mass index; LVEF, left ventricular ejection fraction; VO_2_ peak, oxygen uptake at peak exercise, MBP, mean arterial pressure, HR, heart rate; HFrEF, chronic heart failure; ACEI/ARA, angiotensin-converting enzyme inhibitors/angiotensin II receptor antagonist.*

Spectral parameters of MSNA, RR-interval, SAP, and DAP of HFrEF patients with a lower and higher oscillatory pattern of MSNA are shown in Table 2. The loss of the physiological autonomic modulatory pattern characterized by a paradoxical decrease of LF component in the lower LF_MSNA_/HF_MSNA_ group was observed in the RRi, SAP, and DAP variability. Furthermore, the lower LF_MSNA_/HF_MSNA_ group had a decreased variance of and sympatho-vagal balance (LF/HF) than the higher LF_MSNA_/HF_MSNA_ group had (Table 2). However, the oscillatory component in HF band of RRi and variance of MSNA, SAP, and DAP were similar between groups (Table 2).

**TABLE 2:** Spectral parameters of MSNA, RR-interval, SAP, and DAP of HFrEF patients with lower and higher oscillatory pattern of MSNA.

	Lower LF_MSNA_/HF_MSNA_	Higher LF_MSNA_/HF_MSNA_	*P*
**MSNA**			
Variance, a.u.^2^	0.17 ± 0.02	0.19 ± 0.02	0.49
LF n.u.,%	20 ± 1	70 ± 2	<0.01
HF n.u.,%	72 ± 2	28 ± 2	<0.01
LF/HF	0.3 ± 0.1	4.6 ± 0.9	<0.01
**R-R interval**			
Variance, ms^2^	1828 ± 241	3628 ± 796	0.04
LF n.u.,%	15 ± 2	30 ± 4	<0.01
HF n.u.,%	53 ± 3	49 ± 3	0.44
LF/HF	0.3 ± 0.1	1.4 ± 0.4	<0.01
**Systolic arterial pressure**			
Variance, mmHg^2^	19 ± 3	23 ± 3	0.26
LF abs., mmHg^2^	1.5 ± 0.3	4.8 ± 0.8	<0.001
**Diastolic arterial pressure**			
Variance, mmHg^2^	7.1 ± 0.8	8.4 ± 1.0	0.32
LF abs., mmHg^2^	0.5 ± 0.1	1.7 ± 0.4	<0.001

*Values are mean ± SE; HFrEF, heart failure with reduced ejection fraction; MSNA, muscle sympathetic nerve activity; SAP, systolic arterial pressure; DAP, diastolic arterial pressure; LF_MSNA_/HF_MSNA_, oscillatory pattern of MSNA; LF, low frequency; HF, high frequency; LF/HF, sympatho-vagal balance.*

**FIGURE 1:**
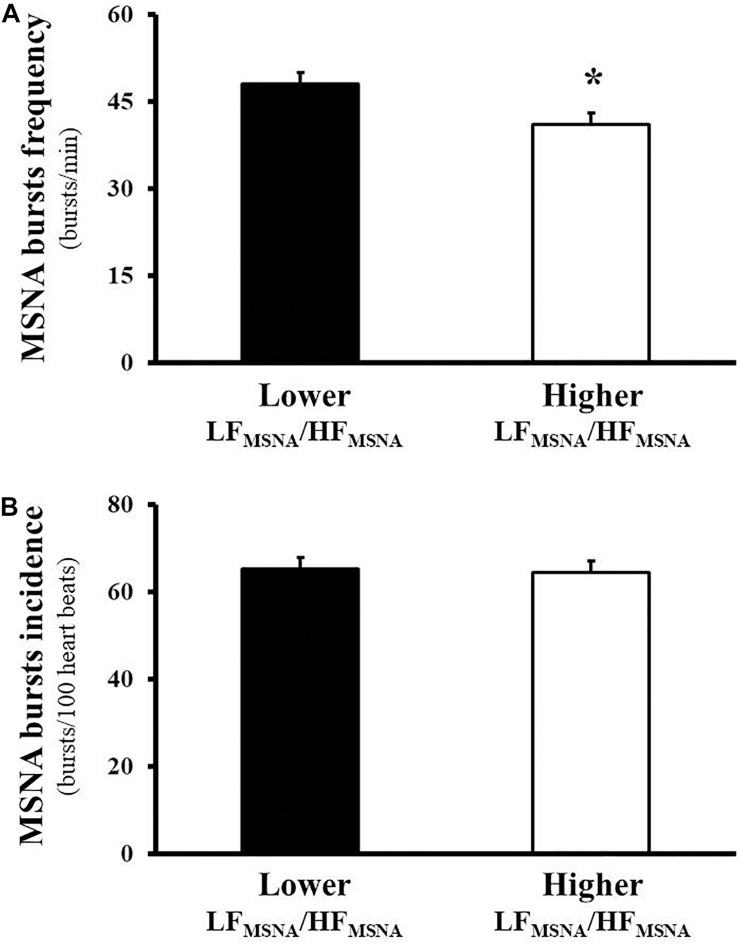
Muscle sympathetic nerve activity (MSNA), assessed directly by the microneurography technique, in heart failure patients with reduced ejection fraction. The tonic activity of the MSNA reveals higher levels in burst frequency (bursts/min, **A**) but not in burst incidence (bursts/100 heart beats, **B**) in the Lower LF_MSNA_/HF_MSNA_ group compared with the Higher LF_MSNA_/HF_MSNA_ group.

In addition, significantly higher levels in burst frequency (Figure 1A) but not in burst incidence of MSNA (Figure 1B) occurred in the lower LF_MSNA_/HF_MSNA_ group compared with the higher LF_MSNA_/HF_MSNA_ group.

**FIGURE 2:**
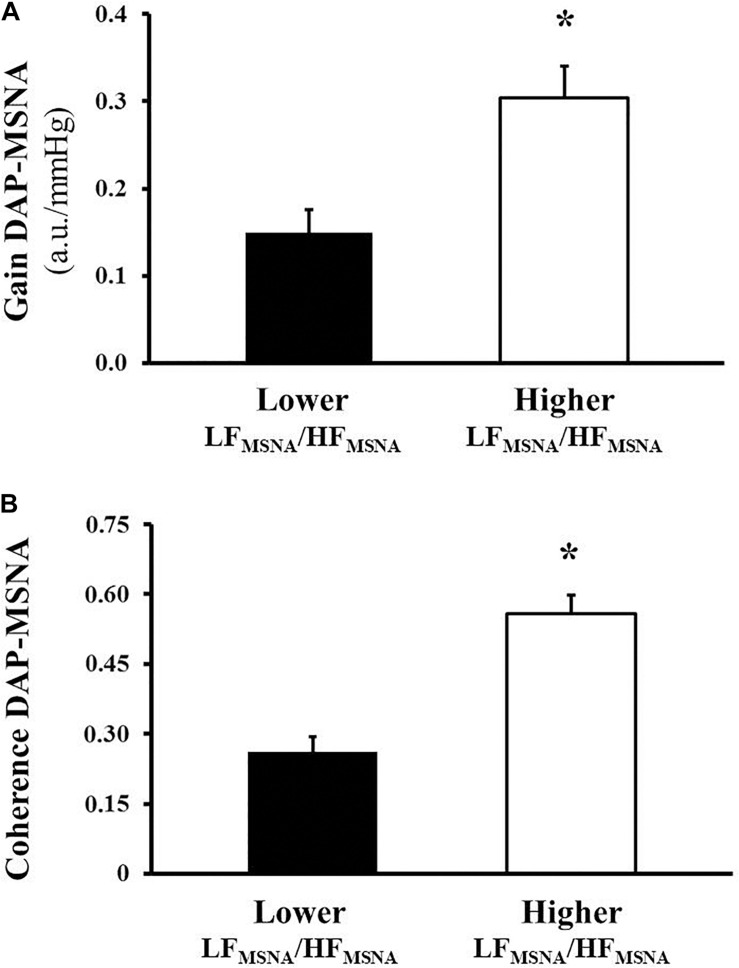
Sympathetic baroreflex function of muscle sympathetic nerve activity (MSNA), assessed by the transfer function technique using bivariate autoregressive spectral analysis, in heart failure patients with reduced ejection fraction. The levels of gain are expressed in arbitrary unit/mm of mercury (u.a./mmHg, **A**) and coherence in percentage **(B)**. *P < 0.01 vs. Group with higher oscillatory pattern of MSNA.

The sympathetic baroreflex function is shown in Figure 2. HFrEF patients with lower LF_MSNA_/HF_MSNA_ had reduced gain and coherence of sympathetic baroreflex function than that observed in patients with higher LF_MSNA_/HF_MSNA_. Further analysis showed that LF_MSNA_/HF_MSNA_ was directly associated with LVEF, gain and coherence of sympathetic baroreflex function, and was inversely associated with HR and MSNA burst frequency (Table 3), yet MSNA burst frequency was significantly associated with HR and inversely associated with gain and coherence of sympathetic baroreflex function (Table 3).

**TABLE 3:** Relationship between oscillatory pattern of MSNA and burst frequency with functional and hemodynamic characteristics and sympathetic baroreflex function in HFrEF patients.

*N* = 93 patients	LF_MSNA_/HF_MSNA_	Burst frequency
LVEF	0.23*	−0.13
VO_2_ peak	0.18	−0.02
HR	–0.46*	0.37*
Gain DAP-MSNA	0.42*	−0.28*
Coherence DAP-MSNA	0.56*	−0.28*
Burst frequency	–0.28*	…

Spearman rank correlation coefficients. HFrEF, heart failure with reduced ejection fraction; LF_MSNA_/HF_MSNA_, oscillatory pattern of muscle sympathetic nerve activity; MSNA, muscle sympathetic nerve activity; LVEF, left ventricular ejection fraction; VO_2_ peak, peak oxygen uptake; HR, heart rate; DAP, diastolic arterial pressure. *P < 0.05.

## Discussion

The main and new findings of the present study were that the oscillatory pattern of MSNA was directly associated with gain and coupling of the sympathetic baroreflex function and inversely associated with MSNA burst frequency in patients with HFrEF. Indeed, our data showed that patients with HFrEF and lower LF_MSNA_/HF_MSNA_ had higher levels of burst frequency and reduced cardiac function, functional capacity, gain and coupling of sympathetic baroreflex function.

In the present study, we aimed to investigate a candidate mechanism underlying the loss of rhythm of the MSNA in HFrEF patients. In this sense, we thought that the reduced gain and coupling of the sympathetic baroreflex function would be involved in this autonomic dysfunction in these patients. Accumulated evidence shows that sympathetic baroreflex plays an important role in the modulation of sympathetic nervous activity (van de Borne et al., 1997; Toschi-Dias et al., 2013). During the cardiac cycle, when blood pressure increases, the activation of arterial baroreceptors located in the aortic arch and in the carotid sinus reflexively restrains the sympathetic efferent outflow. In contrast, during the reduction in arterial pressure when arterial baroreceptors are deactivated the sympathetic activity increases reflexively (Toschi-Dias et al., 2017).

In humans, due to the resonance loop generated in negative feedback mechanisms such as the baroreceptor reflex arc, this rhythm occurs in a modulation range of 0.10 Hz and is produced mainly by the time constant and delays in the local and neuronal phenomena of a closed system (Burgess et al., 2003; Julien, 2006). This autonomic control can be drastically altered in cardiovascular disease. For example, the gain in sympathetic baroreflex is significantly reduced in patients with HFrEF (Grassi et al., 2004), hypertension (Laterza et al., 2007) and myocardial infarction (Martinez et al., 2011). To our knowledge, this is the first time that one of the mechanisms related to the loss of the intrinsic rhythm of sympathetic nervous activity has been demonstrated in patients with HFrEF. In fact, the coupling of the sympathetic baroreflex function is reduced in patients with HFrEF and lower LF_MSNA_/HF_MSNA_.

To the best of our knowledge, few studies have evaluated the phasic activity of neural sympathetic discharge (Pagani et al., 1997; van de Borne et al., 1997; Furlan et al., 2000; Barbic et al., 2015). Most of these studies explore only the oscillatory component in the LF range. Because the MSNA signal acquisition is performed before the adrenergic synapse, the interpretation of the rhythms from the relationship between the spectral components reveals the intrinsic behavior of the sympathetic nervous system. It has been documented that the increase in the LF component of signals involved in cardiovascular variability during physiological stress is the hallmark of phasic sympathetic activity of an effector organ (Furlan et al., 2000; Barbic et al., 2015). Based on the response of oscillatory components during orthostatic stress, an increase of the adrenergic tonic activity is accompanied by a proportional modification of their LF rhythmic pattern of sympathetic firing, RRi, and SAP in healthy subjects.

As mentioned earlier, tonic and phasic activities of the sympathetic firings at rest and during physiological stress occur predominantly at ∼0.10 Hz to ensure appropriate vasoconstriction to organism demand (Pagani et al., 1997; Furlan et al., 2000; Barbic et al., 2015). However, the loss of oscillatory patterns of MSNA, characterized by an increase in burst frequency with the shift from LF range fluctuations toward the HF range, may be observed through the relation of the spectral components of the neural sympathetic discharges in HFrEF patients (van de Borne et al., 1997). This paradoxical phenomenon reveals the saturation of the sympathetic nervous system and is linked to a marked reduction in the gain and coupling of sympathetic baroreflex function. We can speculate that in cardiac dysfunction, as observed in HFrEF patients, some excitatory reflex mechanisms, such as chemoreflex control and cardiac sympathetic afferent reflex, can buffer the baroreflex control leading to a progressive loss in the central autonomic rhythm (Toschi-Dias et al., 2017). Further studies will be necessary to confirm this hypothesis.

Our findings have clinical implications because LF_MSNA_/HF_MSNA_ was also positively associated with LVEF and inversely associated with heart rate. There is a consensus that LVEF is an independent predictor of cardiovascular death, hospitalization, and all-cause mortality (Lewis et al., 2003). Interestingly, it has been demonstrated that patients with HFrEF with an absence of the LF component of MSNA have reduced LVEF and increased hyperadrenergic state (van de Borne et al., 1997). Our findings confirm these observations. We found that the loss of intrinsic rhythm of the MSNA is associated with a worsening in the cardiac systolic function in patients with HFrEF.

The present study extends the knowledge about the influence of the oscillatory pattern of the MSNA on the clinical condition of these patients. These data reveal, for the first time, that HFrEF patients with lower LF_MSNA_/HF_MSNA_ have decreased functional capacity. This finding has clinical implications that should be taken into consideration in medical practice, because it is well established that VO_2_ peak is a prognostic index due to its strong and independent association with clinical outcomes in HFrEF patients (Keteyian et al., 2016). In addition, we demonstrated, in a large population, a positive association between LF_MSNA_/HF_MSNA_ and the levels of MSNA. In addition, we (Barretto et al., 2009) and others (Benedict et al., 1996; Stanek et al., 2001) demonstrated that MSNA and catecholamine levels are independent predictors of mortality in HFrEF patients.

Likewise, of clinical interest, we found that HFrEF patients with lower LF_MSNA_/HF_MSNA_ have increased HR at rest. These data are important because an elevated HR is also considered a prognostic index of mortality (Poole-Wilson et al., 2002, 2003; Kotecha et al., 2017). Our data also suggest that patients with HFrEF under optimized clinical treatment have increased cardiovascular risk in the presence of a decreased MSNA oscillatory pattern.

Our study has some limitations that need to be addressed. First, the experimental protocol considers only recordings obtained with the patient in the resting position to evaluate autonomic nervous system in HFrEF patients. Some authors have suggested that the autonomic nervous system needs to be evaluated both at rest and during physiological maneuvers to examine the complexities of neural regulation. Therefore, it is difficult to generalize our findings to HFrEF patients during a physiological challenge (e.g., exercise, mental stress, and orthostatic maneuvers). Second, even considering that female participants in the present study were a smaller proportion (28 and 19% for the lower and higher LF_MSNA_/HF_MSNA_ groups, respectively), someone could argue about the influence of the menstrual cycle of these women on our results. We did not control the menstrual cycle and the use of hormonal contraceptives or hormone replacement therapy of the women participating in our study. However, because few women were under 50 years of age (i.e., mean age for the end of the reproductive period) our sample was mainly composed of postmenopausal women, who were similarly distributed between the groups. Thus, it is unlikely that menopause, the use of hormonal contraceptives, or hormone replacement therapy could be a confounding variable in the present study. Finally, considering our strategy for the composition of the HFrEF groups, it is possible that some differences in autonomic control markers could be expected due to the interdependence of cardiovascular parameters. Further analysis will be needed in future studies to address this issue.

In conclusion, HFrEF patients with a lower oscillatory pattern of MSNA have a worsening clinical condition as evidenced by the reduced cardiac function and functional capacity, exacerbated resting sympathetic activity, higher resting heart rate, and baroreflex dysfunction when compared with HFrEF patients with a higher oscillatory pattern of MSNA. In addition, there is a direct association between the LF_MSNA_/HF_MSNA_ index and the gain and coupling of sympathetic baroreflex function and the MSNA in HFrEF patients.

## Data Availability Statement

The data, analytic methods, and study materials are available to other researchers at the Heart Institute (InCor) do Hospital das Clínicas da Faculdade de Medicina da Universidade de São Paulo, with Dra. Rondon (urbana@usp.br) or Dr Toschi-Dias (edgar.dias@metodista.br).

## Ethics Statement

The studies involving human participants were reviewed and approved by the Scientific Commission of the Heart Institute (InCor), University of São Paulo Medical School (#3846/13/071) and Human Subject Protection Committee of the Clinical Hospital, University of São Paulo, Medical School (# 22255213.2.0000.0068). The patients/participants provided their written informed consent to participate in this study.

## Author Contributions

ET-D, NM, ET, and MR conceived and designed the research. ET-D, PT, RG, LA-C, TN, DL, AS, LU-P, LM, PO, AB, MA, CN, and MR performed the experiments. ET-D, ET, and NM analyzed the data. ET-D, NM, ET, CN, and MR interpreted the results of the experiments, and edited and revised the manuscript. ET-D and MR prepared the figures. ET-D and ET drafted the manuscript. All authors read and approved the final version of the manuscript.

## Conflict of Interest

The authors declare that the research was conducted in the absence of any commercial or financial relationships that could be construed as a potential conflict of interest.

## Publisher’s Note

All claims expressed in this article are solely those of the authors and do not necessarily represent those of their affiliated organizations, or those of the publisher, the editors and the reviewers. Any product that may be evaluated in this article, or claim that may be made by its manufacturer, is not guaranteed or endorsed by the publisher.
